# Heritability of generalized anxiety stability: a longitudinal twin study among young adults

**DOI:** 10.1017/S0033291725100640

**Published:** 2025-06-30

**Authors:** Julia Funk, Geneviève Morneau-Vaillancourt, Elisavet Palaiologou, Thalia C. Eley

**Affiliations:** 1 Social, Genetic and Developmental Psychiatry Centre, Institute of Psychiatry, Psychology & Neuroscience, King’s College London, London, UK; 2 Department of Psychology, LMU Munich, Munich, Germany; 3 School of Criminology, University of Montreal, Montreal, QC, Canada

**Keywords:** young adulthood, generalized anxiety, twins, longitudinal study, stable genetic influences

## Abstract

**Background:**

Despite the high prevalence of generalized anxiety among young adults, studies investigating factors that shape the course of these symptoms during the twenties are scarce. In addition, generalized anxiety can manifest in different ways, but it is unclear whether symptoms cluster under distinct dimensions in this age group. The current study addressed these gaps using data from the Twins Early Development Study. First, we examined genetic and environmental contributions to continuity and change in generalized anxiety symptoms in young adulthood and the heritability of a latent factor reflecting stability over this period. Next, to explore potential dimensions of generalized anxiety, we investigated the factorial structure of symptoms as well as etiological influences underpinning the different factors.

**Methods:**

The sample comprised 6,429 twin pairs. Generalized anxiety was assessed at six waves (age 23–26 years).

**Results:**

Genetic factors largely accounted for continuity and environmental factors for change in symptom severity. Furthermore, the heritability of stable generalized anxiety (60%) was substantially higher than that at any single time point (39–46%). Regarding the factorial structure of symptoms, we found evidence of two dimensions: worry-avoidance and somatic-distress symptoms. Genetic correlations (*rG* = 0.77–0.91) between the two dimensions were higher than environmental correlations (*rE* = 0.26–0.65).

**Conclusions:**

The current findings suggest that extracting temporal stability provides the strongest opportunity to identify genetic influences on generalized anxiety. Moreover, the results indicate that differences between generalized anxiety dimensions are more likely attributable to environmental than genetic effects.

## Introduction

In recent years, rates of anxiety have risen dramatically among young adults (Archer et al., 2022; Eskander & Bhai, 2023; Goodwin et al., 2020; Slee, Nazareth, Freemantle, & Horsfall, 2021). For instance, a representative survey from 2023 reported that 36% of young adults suffer from generalized anxiety symptoms such as excessive worrying about the future (Eskander & Bhai, 2023). Anxiety is highly disabling (Yang et al., 2021) and particularly when affecting young adults is associated with poorer educational outcomes (Kasteenpohja et al., 2018) and huge costs for economy and society (Hendriks et al., 2015; McDaid & Park, 2022). Given the rising rates of anxiety in young adults as well as the associated burden, it is important to understand etiological factors underlying anxiety during the twenties.

### Temporal stability of anxiety

When investigating the etiology of anxiety, a key aspect to consider is temporal stability. Longitudinal studies show that anxiety symptoms fluctuate over time, both in individuals with and without diagnoses of anxiety disorders (Gustavson et al., 2018; Hovenkamp-Hermelink et al., 2019; Nes et al., 2007; Nivard et al., 2015; Struijs et al., 2020; Waszczuk, Zavos, Gregory, & Eley, 2016). Notably, anxiety symptoms show more temporal fluctuation than other types of psychopathological symptoms (Leopold et al., 2016; Simonoff et al., 2020; Struijs et al., 2020). In addition, anxiety tends to fluctuate more in children, adolescents, and young adults than later in life (Bergen, Gardner, & Kendler, 2007; Nivard et al., 2015; Petkus, Gatz, Reynolds, Kremen, & Wetherell, 2016). However, despite fluctuations, longitudinal studies also show that there is considerable intra-individual stability in anxiety symptoms (Hovenkamp-Hermelink et al., 2019; Nes et al., 2007; Nivard et al., 2015; Prenoveau et al., 2011; Struijs et al., 2020; Waszczuk et al., 2016). As such, most people have a stable tendency reflecting how generally anxious they are.

Distinguishing between stable and time-varying anxiety is crucial as longitudinal twin studies suggest that they are underpinned by somewhat different etiological influences (Garcia et al., 2013; Nivard et al., 2015; Trzaskowski et al., 2012). Specifically, findings indicate that genetic factors largely account for continuity, whereas environmental factors mostly explain change, with studies demonstrating this pattern in childhood (Trzaskowski et al., 2012), adolescence (Garcia et al., 2013; Waszczuk et al., 2016), young adulthood (Nes et al., 2007) as well as over the whole life course (Nivard et al., 2015).

Based on these consistent results, two other longitudinal twin studies in adolescents applied a method that allows to more precisely quantify genetic influences on the stable component of anxiety-like symptoms (Cheesman et al., 2018; Zavos, Gregory, & Eley, 2012). They extracted a latent stability factor from repeated measurements of anxiety sensitivity (Zavos et al., 2012) and emotional problems (Cheesman et al., 2018), respectively, and calculated the heritability of this latent stable factor, which was more heritable in both cases. For example, while genetic factors explained between 33% and 46% of variance in anxiety sensitivity at each of three waves, they explained 61% of variance in stable anxiety sensitivity (Zavos et al., 2012). Notably, extracting stability of emotional problems increased SNP heritability in another study from 5% to 14% (Cheesman et al., 2018). However, despite rising rates of anxiety among young adults (Archer et al., 2022; Goodwin et al., 2020; Slee et al., 2021), no study to date has investigated genetic and environmental contributions to stable anxiety across young adulthood.

### Heterogeneity of anxiety symptoms

Another feature of anxiety is that it can manifest in qualitatively different symptoms (Moriana et al., 2022; Tadi, Pillay, Ejoke, & Khumalo, 2022; Thompson et al., 2021). We recently showed that presentation of generalized anxiety symptoms was associated with age (Thompson et al., 2021). Specifically, younger people felt more irritable and anxious, whereas older people described worrying more. While some studies have examined potential dimensions of symptoms of generalized anxiety during young adulthood, their findings are inconsistent (Byrd-Bredbenner, Eck, & Quick, 2020; Moreno et al., 2019). One found that a two-factor model best explained the structure of generalized anxiety with some items loading on a factor described as cognitive-affective and others on a factor labeled somatic (Moreno et al., 2019). In contrast, another study found support for a model where all items of a generalized anxiety measure loaded on a single factor (Byrd-Bredbenner et al., 2020). More research is needed to identify dimensions of generalized anxiety in young adulthood. In addition to investigating phenotypic dimensions, studies exploring how phenotypic heterogeneity is linked to differences in underlying genetic and environmental factors are crucial.

### Study aims

The current study had three aims. First, we investigated genetic and environmental contributions to continuity and change in generalized anxiety symptom severity in young adulthood, from age 23–26 years. We predicted that genetic factors would substantially contribute to continuity and that environmental factors would largely explain change in generalized anxiety symptoms. Second, given our hypothesis, we examined the heritability of stable generalized anxiety in young adulthood by extracting a latent stability factor across all waves. We predicted that heritability of stable generalized anxiety symptom severity would be higher than heritability of generalized anxiety at any single time point. Third, we explored the factor structure of generalized anxiety in young adulthood to investigate potential symptom dimensions. Due to inconsistencies in the literature, we had no a-priori hypothesis regarding the factor structure. Following identification of dimensions, we tested the extent to which genetic and environmental factors explained their associations. Study aims and hypotheses were preregistered on the Open Science Framework platform (https://osf.io/6j7y5).

## Methods

### Sample

The current study used data from the Twins Early Development Study (TEDS; Lockhart et al., 2023), an ongoing longitudinal study following a cohort of twins born in England and Wales between 1994 and 1996. For the purpose of this study, we used data from the most recent six waves of assessment. Waves 2–5 took place during the Covid-19 pandemic. The sample for the analyses comprised 6,429 twin pairs, where at least one twin had data for at least one wave (10,836 individuals with data for at least one wave). Of this sample, 2,199 twin pairs were monozygotic (MZ) and 4,230 were dizygotic (DZ). Fifty-eight percent of the sample was female. Mean age (and standard deviation) was 22.85 (0.88) years at wave 1, 24.85 (0.85) years at wave 2, 25.02 (0.86) years at wave 3, 25.33 (0.86) years at wave 4, 25.73 (0.86) years at wave 5, and 26.38 (0.91) years at wave 6. Ethical approval for TEDS was granted by the King’s College London Ethics Committee. Prior to each assessment wave, informed consent was collected.

### Measures

Generalized anxiety was assessed via the Generalized Anxiety Disorder – Dimensional measure (GAD-D; Lebeau et al., 2012). The GAD-D is a 10-item self-report measure of the severity of generalized anxiety symptoms. Respondents are asked to rate items, such as ‘I have felt anxious, worried, or nervous’ on a 5-point scale ranging from 0 ‘never’ to 4 ‘all of the time’. Total scale scores range from 0 to 40. The measure has demonstrated good psychometric properties (Lebeau et al., 2012). Originally, the measure was designed to assess the severity of symptoms with respect to the past months; however, the current study used an adapted version that assesses symptoms with respect to the past week (Craske et al., 2013). Internal consistency of the GAD-D in the current study was high at all waves (*α* = 0.91–0.92).

### Statistical analyses

The current study used twin models to estimate genetic and environmental contributions to generalized anxiety symptom severity (total score on the GAD-D). The twin design compares similarities between MZ twin pairs that share 100% of their genes and DZ twin pairs that share on average 50% of their genes. Based on differences in within-pair correlations across MZ and DZ twins, it is possible to estimate the influences of additive genetic effects (A), dominance genetic effects (D), shared environmental effects (C), and non-shared environmental effects (E) (Plomin et al., 2013). We estimated univariate twin models to assess the extent to which generalized anxiety at each of the six waves can be explained by genetic and environmental factors. We then conducted multivariate twin models to estimate genetic and environmental effects on generalized anxiety across waves. We first investigated genetic and environmental contributions to continuity and change in generalized anxiety using a Cholesky decomposition. In this model, contributions to continuity are quantified by estimating the extent to which genetic and environmental effects on symptoms at earlier waves influence symptoms at later waves. The Cholesky decomposition also estimates the magnitude of time-specific genetic and environmental effects (i.e. change). Second, to examine the heritability of stable generalized anxiety, we used a common pathway model. This model estimates the extent to which symptoms at each wave are explained by a latent stability factor and quantifies genetic and environmental influences on this latent stability factor and on the time-specific components. For univariate and multivariate twin analyses, we estimated ACE, ADE, and AE models and interpreted the model with the best fit. We conducted all twin analyses using the R package ‘OpenMx’ (Neale et al., 2016). Missing data were handled via Full Information Maximum Likelihood (FIML) in the twin models.

In addition, we conducted factor analyses to investigate potential dimensions of generalized anxiety. To prevent relatedness from confounding results on the phenotypic structure of generalized anxiety, co-twins were randomly excluded. At each wave, an exploratory factor analysis was performed in a randomly selected 70% subset of the data using the R package ‘psych’ (Revelle, 2017). Minimum item loading was defined as the minimum number of items loadings >0.3 and greater than on any other factors. Subsequently, we conducted confirmatory factor analyses at each wave in the remaining 30% of the data to test the factor structure derived by the exploratory factor analyses using the r package ‘lavaan’ (Rosseel, 2012). We then repeated the confirmatory factor analyses based on the whole sample at each wave to provide overview fit statistics. Missing data were handled via pairwise deletion in the exploratory factor analyses and via FIML in the confirmatory factor analyses. Evidence for dimensions was defined as follows: (1) Exploratory factor analyses showing a consistent factor structure with more than one factor across all waves and (2) confirmatory factor analyses demonstrating good model fit for this factor structure at all waves. In case of evidence for dimensions, we planned to rerun the univariate and multivariate twin models separately for the single dimensions. In addition, we planned to analyze genetic and environmental contributions to overlap and specificity of dimensions by estimating a correlated-factor model.

## Results

### Phenotypic correlations

Table 1 shows descriptive statistics for generalized anxiety scores at each of the six waves as well as longitudinal correlations. Mean scores were slightly higher for the waves that were conducted during the Covid-19 pandemic (waves 2–5). Generalized anxiety showed moderate-to-high temporal stability (*r* = 0.56–0.76). Correlations were smaller the higher the temporal distance between assessment waves.

**Table 1.** Descriptive statistics for generalized anxiety scores at each wave and longitudinal correlations (with 95% confidence intervals)

	*N* (n_MZ_/n_DZ_)	Age	*M*	*SD*	1	2	3	4	5
1. Generalized anxiety wave 1	3340 (1341/1999)	22.85	7.35	7.46					
2. Generalized anxiety wave 2	1484 (675/809)	24.85	8.63	7.52	0.57 (0.55–0.59)				
3. Generalized anxiety wave 3	1218 (541/677)	25.02	8.82	7.71	0.59 (0.56–0.61)	0.75 (0.74–0.77)			
4. Generalized anxiety wave 4	1093 (510/583)	25.33	9.26	7.95	0.57 (0.54–0.59)	0.69 (0.68–0.71)	0.73 (0.71–0.75)		
5. Generalized anxiety wave 5	1143 (539/604)	25.73	8.81	7.65	0.57 (0.55–0.59)	0.69 (0.67–0.71)	0.72 (0.70–0.74)	0.76 (−0.74–0.77)	
6. Generalized anxiety wave 6	2808 (1137/1671)	26.38	7.77	7.35	0.56 (0.54–0.57)	0.63 (0.61–0.65)	0.66 (0.64–0.68)	0.68 (0.66–0.69)	0.71 (0.69–0.73)

*Note:* Generalized anxiety waves 1–6 = raw total score on the GAD-D, waves 1–6, *N* (n_MZ_/n_DZ_) = number of twin pairs with data for both twins per wave, total number is printed before brackets, numbers of monozygotic (n_MZ_), and dizygotic (n_DZ_) twin pairs are printed within brackets), age = mean age in years at each wave. Waves 2–5 took place during the Covid 19 pandemic.

### Univariate twin models

Due to skewness, GAD-D total scale scores were square root transformed before twin analysis. The best-fitting univariate twin models were an ADE model for generalized anxiety at wave 1 and AE models for generalized anxiety at waves 2–6. Fit statistics for all univariate twin models at each wave can be found in the Supplementary Information (Table S1). In the univariate models, variance explained by genetic factors ranged from 39% to 46% (see Supplementary Information, Table S2).

### Multivariate twin models

#### Cholesky decomposition

**Figure 1.**
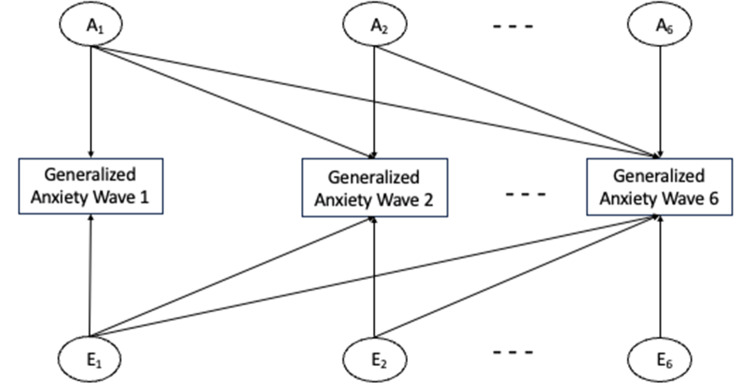
Cholesky decomposition of generalized anxiety. A = additive genetic factors; E = non-shared environmental factors. The figure only includes three of the six waves, but the model was estimated based on six waves. Dashes represent wave 3 to 5.

The best-fitting Cholesky model was an AE model, see Table 2 for fit statistics and Figure 1 and Table 3 for the model and parameter estimates. As predicted, the model showed that genetic factors largely contributed to continuity in generalized anxiety from wave 1–6; the first set of genetic factors (A_1_) accounted for 39% of the variance at wave 1 and continued to account for a substantial part of the variance at the later waves (34–38%). There also was some evidence of genetic innovation, mostly at wave 2, but even if significant, effects of later genetic factors were considerably smaller than effects of the first set of genetic factors. In line with our expectations, environmental effects were largely time-specific, with time-specific environmental factors explaining between 31% and 61% of variance at each wave, but there was also some environmental continuity. For example, the first set of environmental factors (E1) continued to have significant effects on generalized anxiety at later waves (accounting for 7–9% of variance at waves 2–6).

**Table 2.** Fit comparisons for multivariate twin models of generalized anxiety

Base model	Comparison model	−2LL	df	AIC	Δ −2LL	Δ df	*p*
Saturated	–	97392.66	32794	97752.66	N/A	N/A	N/A
Saturated	Constrained	97539.09	32905	97677.09	146.44	111	<0.05
*Cholesky decomposition*							
Saturated	ACE	97549.26	32905	97687.26	156.60	111	<0.01
Saturated	AE	97550.97	32926	97646.97	158.32	132	0.06
Saturated	ADE	97545.35	32905	97683.35	152.69	111	<0.01
ACE	AE	97550.97	32926	97646.97	1.71	21	1
ADE	**AE**	**97550.97**	**32926**	**97646.97**	**5.62**	**21**	**1**
*Common pathway*							
Saturated	ACE	97724.65	32942	97790.65	331.00	148	<0.001
Saturated	AE	97724.70	32949	97776.70	332.04	155	<0.001
Saturated	ADE	97721.67	32942	97787.67	329.01	148	<0.001
ACE	AE	97724.70	32949	97776.70	0.04	7	1
ADE	**AE**	**97724.70**	**32949**	**97776.70**	**3.02**	**7**	**0.88**

*Note:* −2LL = minus twice the log likelihood; df = degrees of freedom; AIC = Akaike’s information criterion; A = additive genetic factors; C = shared environmental factors; E = non-shared environmental factors; D = dominance genetic factors; constrained = constrained model with equal means and variances across twins and zygosity groups as well as symmetric cross-twin cross-wave covariance matrices. The best-fitting Cholesky and common pathway models are indicated in bold. Fit of the best-fitting common pathway model was significantly worse than fit of the saturated model. However, this is common in twin studies with large sample sizes, where minimal variance deviations from the model’s assumptions can be statistically significant (e.g. Cheesman et al., 2018; Waszczuk et al., 2016).

**Table 3.** Parameter estimates with 95% confidence intervals for the Cholesky decomposition of generalized anxiety

	Wave 1 factors		Wave 2 factors		Wave 3 factors		Wave 4 factors		Wave 5 factors		Wave 6 factors	
	A_1_	E_1_	A_2_	E_2_	A_3_	E_3_	A_4_	E_4_	A_5_	E_5_	A_6_	E_6_
Generalized anxiety wave 1	**0.39** (0.35–0.43)	**0.61** (0.57–0.56)										
Generalized anxiety wave 2	**0.34** (0.28–0.39)	**0.09** (0.07–0.11)	**0.11** (0.06–0.15)	**0.46** (0.43–0.51)								
Generalized anxiety wave 3	**0.36** (0.30–0.41)	**0.09** (0.07–0.12)	**0.05** (0.01–0.09)	**0.13** (0.12–0.17)	0.01 (0–0.03)	**0.36** (0.33–0.39)						
Generalized anxiety wave 4	0.**38** (0.32–0.44)	**0.08** (0.06–0.10)	**0.04** (0.01–0.09)	**0.10** (0.07–0.13)	0.01 (0–0.06)	**0.06** (0.04–0.07)	0.02 (0.00–0.05)	**0.32** (0.29–0.35)				
Generalized anxiety wave 5	**0.36** (0.30–0.42)	**0.08** (0.06–0.11)	**0.07** (0.03–0.12)	**0.07** (0.05–0.09)	0 (0–0.05)	**0.05** (0.03–0.07)	0.02 (0–0.04)	**0.04** (0.02–0.05)	0 (0–0.03)	**0.31** (0.29–0.34)		
Generalized anxiety wave 6	**0.36** (0.32–0.41)	**0.07** (0.05–0.09)	**0.03** (0.01–0.07)	**0.05** (0.03–0.07)	0 (0.00–0.09)	**0.03** (0.02–0.05)	0.03 (0–0.09)	**0.02** (0.01–0.03)	0 (0–0.07)	**0.02** (0.01–0.03)	0.02 (0–0.06)	**0.36** (0.33–0.39)

*Note:* A = additive genetic factors; E = non-shared environmental factors; generalized anxiety waves 1–6 = square root transformed total score on the GAD-D, waves 1–6. Parameter estimates presented in the table are variance components. To obtain path coefficients, estimates should be square rooted. Significant estimates are indicated in bold.

#### Common pathway model

**Figure 2.**
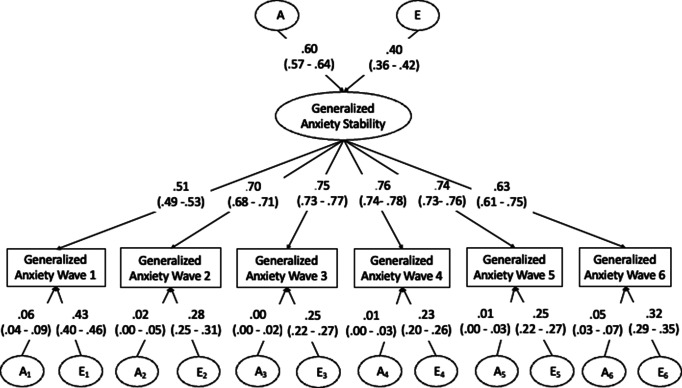
Common pathway model of generalized anxiety. A = additive genetic factors; E = non-shared environmental factors; generalized anxiety wave 1 to 6 = square root transformed total score on the GAD-D, wave 1 to 6; generalized anxiety stability = latent stability of generalized anxiety. Parameter estimates presented in the figure are variance components. To obtain path coefficients, estimates should be square rooted.

The best-fitting common pathway model was an AE model, see Table 2 for fit statistics and Figure 2 for the model with parameter estimates. The latent stability factor explained between 51% and 76% of the phenotypic variance in generalized anxiety at the single waves. Consistent with our expectations, genetic factors explained more variance in stable generalized anxiety (60%) than in generalized anxiety at any single time point (39–46%). In line with the results of the Cholesky decomposition, time-specific variance in generalized anxiety was largely explained by environmental factors (23–43%).

### Phenotypic dimensions of generalized anxiety

Exploratory factor analyses at each wave consistently showed that a two-factor structure fit the data best (see Supplementary Information, Table S3). Factor loadings largely showed the same pattern for all waves (see Supplementary Information, Table S4). At five waves, six items loaded highest on factor 1, which we labeled ‘somatic-distress’ based on item content, and three items loaded highest on factor 2, which labeled ‘worry-avoidance’. At wave 3, one additional item loaded highest on the worry-avoidance factor. To test the factor structure further, we performed confirmatory factor analyses at each wave. For wave 3, we additionally tested a model with the same pattern of factor loadings as in the models at the other waves. At each of the waves, the model fit of the two-factor model with six items loading on the somatic-distress factor and three items loading on the worry-avoidance factor was good (see Supplementary Information, Table S5).

#### Twin models of generalized anxiety dimensions

Univariate twin models showed that genetic factors accounted for 39–47% of variance in somatic-distress symptoms and for 29–37% of variance in worry-avoidance symptoms (see Supplementary Information, Tables S6–7 and S10–11, for fit statistics and parameter estimates of the models). Cholesky decompositions yielded similar patterns as for generalized anxiety total scale scores with genetics largely accounting for continuity and environmental factors for change in both symptom subtypes (see Supplementary Information, Tables S9–S13). Common pathway models showed that heritability of stable somatic distress (55%) and heritability of stable worry avoidance (62%) were substantially higher than heritability of these symptoms at the single time points (see Supplementary Information, Figures S1 and S2). The latent stability factor explained more variance in the somatic distress (51–75%) than in the worry-avoidance dimension (39–64%). In addition, heritability estimates for somatic-distress symptoms were slightly higher than for worry-avoidance symptoms; however, these differences are negligible as estimates for the two dimensions were still within each other’s confidence intervals. A correlated-factor model (see Supplementary Information, Table S14 and Figure S3) demonstrated that genetic correlations between somatic-distress and worry-avoidance symptoms were high (0.90–0.91 at the same time point and 0.77–0.85 cross time point). Environmental correlations between the symptom subtypes were lower than genetic correlations but still in the moderate range (0.62–0.65 at the same time point and 0.26–0.28 cross time point).

## Discussion

We found that genetic influences are the primary factor contributing to continuity in generalized anxiety in young adulthood. Furthermore, the heritability of a latent factor reflecting stability of generalized anxiety was substantially higher (60%) than heritability at any single time point (39–46%). In addition, although symptoms phenotypically clustered under a somatic-distress and a worry-avoidance dimension, underlying genetic influences were largely shared. In contrast, environmental factors showed more specificity to assessment time point and symptom dimension.

### Phenotypic stability of generalized anxiety

Results indicated that despite fluctuations, generalized anxiety has a stable core in young adulthood. Interestingly, the latent stability factor explained more variance for the waves where mean generalized anxiety was higher. This finding fits with evidence from a daily diary study in patients with Generalized Anxiety disorder suggesting that higher mean anxiety symptoms severity is associated with less fluctuation in symptom severity (Fisher & Newman, 2016). In addition, the respective latent stability factor explained more variance in the somatic distress (51–75%) than in the worry-avoidance dimension (39–64%), suggesting that the latter might be more susceptible to change. Consistent with this, accumulating evidence suggests that repetitive negative thinking such as worrying responds well to different psychological interventions (Bell et al., 2023; Monteregge, Tsagkalidou, Cuijpers, & Spinhoven, 2020). The finding that generalized anxiety has stable as well as dynamic components in young adulthood highlights the importance of considering persistent as well as time-specific etiological influences.

### Factors underlying continuity and change of generalized anxiety from wave to wave

In line with prior studies (Garcia et al., 2013; Nes et al., 2007; Nivard et al., 2015; Trzaskowski et al., 2012; Waszczuk et al., 2016), genetic factors largely contributed to continuity in generalized anxiety across the study period. Specifically, the first set of genetic factors not only explained variance in generalized anxiety at age 21 but also continued to substantially impact generalized anxiety into the mid-twenties. In addition, new genetic influences emerged at wave 2 (age ~25), the first wave conducted during the pandemic and continued to have small, but significant effects on generalized anxiety at the later waves. No other new genetic effects were seen at any time point; thus, genetic effects were somewhat less dynamic than in childhood and adolescence (Kendler, Gardner, & Lichtenstein, 2008; Waszczuk et al., 2016; Zavos et al., 2012). Similar to studies in other age groups, environmental effects were largely time-specific, indicating that environmental influences drive change in generalized anxiety during young adulthood. In contrast to studies in children and adolescents (Garcia et al., 2013; Kendler et al., 2008; Trzaskowski et al., 2012; Waszczuk et al., 2016), results furthermore indicated small but persistent environmental effects on generalized anxiety. These sustained environmental effects might contribute to the increased prevalence of generalized anxiety among young adults relative to other age groups (Eskander & Bhai, 2023). Environmental factors that increase the risk for persistently heightened levels of anxiety during young adulthood could, for example, be difficulties arising from the transition between education and employment (Klug, Drobnič, & Brockmann, 2019).

### Factors explaining the stable component of generalized anxiety

To better quantify stable effects on generalized anxiety in young adulthood, we tested the extent to which variance in stable generalized anxiety was explained by genetic and environmental factors. In line with prior studies in adolescence (Cheesman et al., 2018; Zavos et al., 2012), the heritability of stable generalized anxiety was substantially higher (60%) than that for any single time point (39–46%). The increased heritability of stable relative to time-specific anxiety has important implications for genomic studies of anxiety. Genome-wide association studies have identified DNA variations associated with anxiety (Levey et al., 2020; Meier et al., 2019; Purves et al., 2020). However, as anxiety is only moderately heritable and highly polygenic, large sample sizes are required to detect associations (Smoller, 2020). Using a latent stable anxiety factor may help to overcome these challenges and provides a strong opportunity for identifying DNA variations contributing to the heritability of anxiety.

Our findings also have implications for studies of environmental influences on anxiety. Prior studies largely identified environmental influences as being time-specific (Garcia et al., 2013; Kendler et al., 2008; Nivard et al., 2015; Trzaskowski et al., 2012; Waszczuk et al., 2016). However, in the current study, we found that environmental factors explained 40% of variance in stable generalized anxiety. Extracting stability likely increases the accuracy of the measure and thus potentially increases the power to detect all types of risk factors, whether genetic or environmental.

### Etiological overlap and specificity of generalized anxiety dimensions

Generalized anxiety symptoms in young adults were also found to reflect two factors: somatic distress and worry avoidance. However, given that genetic influences on somatic-distress and worry-avoidance symptoms were largely shared, for genetic studies at least, there is no need to examine these specific dimensions. In contrast, environmental influences were more specific to specific symptom dimensions. As such, when examining the effects of environmental risk factors on generalized anxiety (e.g. low socioeconomic status; Moffitt et al., 2007), it may be useful to utilize measures assessing these two dimensions.

### Potential influence of the Covid-19 pandemic

The fact that four of the six waves took place during the Covid-19 pandemic raises the question of whether the pandemic influenced the results. On the one hand, neither the phenotypic structure of generalized anxiety nor the overall magnitude of genetic and environmental influences differed significantly between pandemic and non-pandemic waves. Correlations between consecutive waves during and after the pandemic (waves 2–6) were stronger than those between the pre-pandemic wave and wave 2, but this could be explained by their higher temporal distance compared to gaps between all other consecutive waves. On the other hand, mean anxiety scores were higher, and the latent stability factor accounted for more variance in waves conducted during the pandemic. These findings suggest that systematic environmental stressors during the Covid-19 pandemic, such as recurring lockdowns (Panchal et al., 2023; Pieh et al., 2021), led to persistently heightened levels of anxiety during this period. In addition, it is noteworthy that only in the pre-pandemic wave did the ADE model show a better fit than the AE model, suggesting dominant genetic effects, and that genetic innovation was observed only in the first pandemic wave. Although these two findings do not clearly differ between pandemic and non-pandemic waves, replication is needed to determine whether they result from age-specific genetic effects or were influenced by the pandemic.

### Limitations

The current study has some limitations. First, while it is a strength having six waves, the design was limited by varying temporal distance between the waves. Specifically, the gap between the first and the second wave was relatively large (2 years), whereas the time interval between the remaining waves was shorter (approximately 3–6 months). Therefore, it is unclear whether the evidence for genetic innovation at wave 2 is specific to age 25 or results from the temporal distance between the first and second wave. Second, the generalizability of the results may be limited by the collection of much of the data during and shortly after the Covid-19 pandemic. Considering that the latent stability factor explained more variance in waves that were conducted during the pandemic, it is crucial to replicate the findings with sufficient temporal distance to the pandemic. Reassuringly, however, the overall pattern of stable and dynamic influences on anxiety found in the current study is in line with earlier studies in younger age groups (Cheesman et al., 2018; Zavos et al., 2012). In addition, we used the GAD-D (Lebeau et al., 2012) for assessing generalized anxiety. A more commonly used measure of generalized anxiety is the Generalized Anxiety Disorder Questionnaire-7 (GAD-7; Spitzer, Kroenke, Williams, & Löwe, 2006). Even though the item content of the GAD-D and GAD-7 is similar, small differences, e.g., in the number of items, limit the comparability of the current findings with earlier studies. However, the greater length of this scale may have supported the identification of the two sub-scales, for which prior evidence using the GAD7 was mixed (Byrd-Bredbenner et al., 2020; Moreno et al., 2019). Finally, we only assessed generalized anxiety from early to mid-twenties. Our study revealed some differences to prior findings on the course of generalized anxiety in younger age groups, i.e., less dynamic genetic effects and more persistent environmental effects. However, to draw definitive conclusions about what drives the rise of generalized anxiety in the twenties, future studies should assess generalized anxiety covering an extended period from the teenage years to early thirties.

## Conclusions and future directions

The current findings suggest that extracting temporal stability of anxiety could substantially increase power for genomic studies of anxiety. In addition, we found that environmental factors have persistent effects on generalized anxiety in young adulthood, which has implications for investigating how environmental risks shape anxiety during this period. Exploring whether putative environmental risk factors have short- or long-term effects or differentially impact symptom dimensions could bring new insights into the etiology of generalized anxiety in young adulthood and ultimately inform treatment development.

## Supporting information

Funk et al. supplementary material Funk et al. supplementary material

## Supplementary material

The supplementary material for this article can be found at http://doi.org/10.1017/S0033291725100640.
