# Supplementary material for: Heritability of generalized anxiety stability: a longitudinal twin study among young adults
Source: Psychol Med. 2025 Jun 30;55:e180. doi: 10.1017/S0033291725100640 (PMC12234016; doi:10.1017/S0033291725100640)
Supplement: Funk et al. supplementary material [file S0033291725100640sup001.docx]

**Supporting Information**

| **A. Univariate twin models of generalized anxiety**  **Table S1**  *Fit comparisons for univariate twin models of generalized anxiety* | | | | | | | |
| --- | --- | --- | --- | --- | --- | --- | --- |
| Base Model | Comparison Model | -2LL | df | AIC | Δ -2LL | Δ df | *p* |
| *Generalized anxiety wave 1* | | | | | | | |
| Saturated | - | 29171.34 | 8395 | 29191.34 | N/A | N/A | N/A |
| Saturated | ACE | 29181.31 | 8401 | 29189.31 | 9.97 | 6 | .13 |
| ACE | AE | 29181.31 | 8402 | 29187.31 | 0.00 | 1 | 1 |
| Saturated | **ADE** | **29177.37** | **8401** | **29185.37** | **6.04** | **6** | **.42** |
| ADE | AE | 29181.31 | 8402 | 29187.31 | 3.93 | 3.93 | <.05 |
| *Generalized anxiety wave 2* | | | | | | | |
| Saturated | - | 16449.24 | 4875 | 16469.24 | N/A | N/A | N/A |
| Saturated | ACE | 16455.82 | 4881 | 16463.82 | 6.58 | 6 | .36 |
| ACE | **AE** | **16455.82** | **4882** | **16461.82** | **0.00** | **1** | **1** |
| Saturated | ADE | 16455.74 | 4881 | 16463.74 | 6.50 | 6 | .37 |
| ADE | AE | 16455.82 | 4882 | 16461.82 | 0.08 | 1 | .78 |
| *Generalized anxiety wave 3* | | | | | | | |
| Saturated | - | 13787.01 | 4028 | 13807.01 | N/A | N/A | N/A |
| Saturated | ACE | 13797.17 | 4034 | 13805.17 | 10.17 | 6 | .12 |
| ACE | **AE** | **13797.65** | **4035** | **13803.65** | **0.47** | **1** | **.49** |
| Saturated | ADE | 13797.65 | 4034 | 13805.65 | 10.64 | 6 | .10 |
| ADE | AE | 13797.65 | 4035 | 13803.65 | 0.00 | 1 | 1 |
| *Generalized anxiety wave 4* | | | | | | | |
| Saturated | - | 12507.03 | 3638 | 12527.03 | N/A | N/A | N/A |
| Saturated | ACE | 12516.98 | 3644 | 12524.98 | 9.96 | 6 | .13 |
| ACE | **AE** | **12516.98** | **3645** | **12522.98** | **0.00** | **1** | **1** |
| Saturated | ADE | 12516.26 | 3644 | 12524.26 | 9.23 | 6 | .16 |
| ADE | AE | 12516.98 | 3645 | 12522.98 | 0.73 | 1 | .39 |
| *Generalized anxiety wave 5* | | | | | | | |
| Saturated | - | 13214.33 | 3863 | 13234.33 | N/A | N/A | N/A |
| Saturated | ACE | 13223.07 | 3869 | 13231.07 | 8.74 | 6 | .19 |
| ACE | **AE** | **13223.07** | **3870** | **13229.07** | **0.00** | **1** | **1** |
| Saturated | ADE | 13223.05 | 3869 | 13231.05 | 8.72 | 6 | .19 |
| ADE | **AE** | **13223.07** | **3870** | **13229.07** | **0.02** | **1** | **.89** |
| *Generalized anxiety wave 6* | | | | | | | |
| Saturated | - | 28144.65 | 8115 | 28164.65 | N/A | N/A | N/A |
| Saturated | ACE | 28151.64 | 8121 | 28159.64 | 6.99 | 6 | .32 |
| ACE | **AE** | **28151.64** | **8122** | **28157.64** | **0.00** | **1** | **1** |
| Saturated | ADE | 28150.61 | 8121 | 28158.61 | 5.96 | 6 | .43 |
| ADE | AE | 28151.64 | 8122 | 28157.64 | 1.02 | 1 | .31 |
| *Note.* -2LL = minus twice the log likelihood; df = degrees of freedom; AIC = Akaike’s information criterion; A = additive genetic factors; C= shared environmental factors; E = non-shared environmental factors; D = dominance genetic factors. The best fitting genetic model is indicated in bold. | | | | | | | |

|  | rMZ | rDZ | A | D | E |
| --- | --- | --- | --- | --- | --- |
| Generalized anxiety wave 1 | .41  (.36 - .45) | .15  (.11 - .19) | .22  (.04 - .39) | .19  (.01 - .39) | .59  (.55 - .63) |
| Generalized anxiety wave 2 | .44  (.38 - .50) | .22  (.15 - .28) | .45  (.40 - .59) | - | .55  (.50 - .60) |
| Generalized anxiety wave 3 | .38  (.30 - .45) | .20  (.13 - .28) | .39  (.35 - .43) | - | .61  (57 - .65) |
| Generalized anxiety wave 4 | .47  (.40 - .53) | .20  (.12 - .27) | .46  (.40 - .52) | - | .54  (.48 - .60) |
| Generalized anxiety wave 5 | .44  (.37 - .51) | .23  (.15 - .30) | .45  (.39 - .51) | - | .55  (.49 - .61) |
| Generalized anxiety wave 6 | .45  (.40 - .49) | .19  (.14 - .24) | .44  (.40 - .48) | - | .56  (.52 - .60) |

**Table S2**

*Twin correlations and parameter estimates with 95% confidence intervals for univariate twin models of generalized anxiety*

*Note.* rMZ = cross-twin correlations for monozygotic twins; rDZ = cross-twin correlations for dizygotic twins; A = additive genetic factors; D = dominance genetic factors; E = non-shared environmental factors; generalized anxiety wave 1 to 6 = square root transformed total score on the GAD-D, wave 1 to 6. Parameter estimates presented in the table are variance components. To obtain path coefficients, estimates should be square rooted.

**B. Phenotypic dimensions of generalized anxiety – factor analyses**

**Table S3**

*Model fit statistics for exploratory factor analysis of* *generalized anxiety items*

| Number of factors | | df | RMSEA (≤.05) | RMSEA 90% CI | TLI  (≥.95) | BIC | SRMR (≤.08) | Cumulative variance | Minimum item loading |
| --- | --- | --- | --- | --- | --- | --- | --- | --- | --- |
| *wave 1* | | |  |  |  |  |  |  |  |
| 1 | | 35 | .124 | (.120 - .127) | .908 | 2924.9 | .04 | .62 | 10 |
| **2** | | **26** | **.078** | **(.074 - .083)** | **.963** | **751.16** | **.02** | **.67** | **3** |
| 3 | | 18 | .049 | (.044 - .054) | .986 | 120.34 | .01 | .69 | 1 |
| *wave 2* | | |  |  |  |  |  |  |  |
| 1 | 35 | | .128 | (.123 - .133) | . 897 | 1726.61 | .05 | .60 | 10 |
| **2** | **26** | | **.084** | **(.078 - .089)** | **.956** | **445.33** | **.02** | **.65** | **3** |
| 3 | 18 | | .060 | (.053 - .067) | .978 | 92.41 | .01 | .68 | 1 |
| *wave 3* |  | |  |  |  |  |  |  |  |
| 1 | 35 | | .124 | (.118 - .129) | .909 | 1254.83 | .04 | .62 | 10 |
| **2** | **26** | | **.083** | **(.077 - .090)** | **.958** | **327.35** | **.02** | **.66** | **4** |
| 3 | 18 | | .047 | (.039 - .054) | .987 | -15.69 | .01 | .69 | 1 |
| *wave 4* |  | |  |  |  |  |  |  |  |
| 1 | 35 | | .124 | (.118 - .130) | .908 | 1140.56 | .04 | .62 | 10 |
| **2** | **26** | | **.074** | **(.068 - .081)** | **.967** | **187.11** | **.02** | **.66** | **3** |
| 3 | 18 | | .055 | (.047 - .063) | .982 | 14.6 | .01 | .69 | 0 |
| *wave 5* |  | |  |  |  |  |  |  |  |
| 1 | 35 | | .134 | (.129 - .140) | .888 | 1494.58 | .05 | .61 | 10 |
| **3** | **26** | | **.075** | **(.069 - .081)** | **.965** | **222.18** | **.02** | **.66** | **3** |
| 3 | 18 | | .052 | (.045 - .060) | .983 | 11.74 | .01 | .68 | 1 |
| *wave 6* |  | |  |  |  |  |  |  |  |
| 1 | 35 | | .143 | (.139 - .147) | .880 | 3871.57 | .05 | .62 | 10 |
| **2** | **26** | | **.079** | **(.075 - .083)** | **.963** | **740.68** | **.02** | **.67** | **3** |
| 3 | 18 | | .060 | (.055 - .065) | .979 | 235.93 | .01 | .69 | 0 |

*Note*. Generalized anxiety items = items of the GAD-D; BIC = Bayesian information criterion; df = degrees of freedom; RMSEA = root mean square error of approximation; SRMR = standardized root mean square residuals; TLI = Tucker–Lewis fit index. The cut off for good fit for each fit index is printed in the header. For BIC, the lowest BIC relative to the other models indicates the best fit. Cumulative variance is defined as cumulative proportion of variance explained by all factors. Minimum item loading is defined as the minimum number of items loadings >.3 and greater than on any other factors. Factors were allowed to correlate using oblimin rotation. Best fitting and chosen models for each wave are indicated in bold.

**Table S4**

*Factor loadings for the two factor solutions of generalized anxiety items*

| Item | wave 1 | | wave 2 | | wave 3 | | wave 4 | | wave 5 | | wave 6 | | |
| --- | --- | --- | --- | --- | --- | --- | --- | --- | --- | --- | --- | --- | --- |
|  | F1 | F2 | F1 | F2 | F1 | F2 | F1 | F2 | F1 | F2 | F1 | F2 |  |
| I have felt moments of sudden terror, fear, or fright | .84 | .05 | .89 | -.06 | .90 | -.06 | .91 | -.09 | .90 | -.08 | .88 | -.02 |  |
| I have felt anxious, worried, or nervous | .73 | .16 | .83 | .07 | .70 | .21 | .87 | .01 | .81 | .08 | .80 | .03 |  |
| I have had thoughts of bad things happening, such as family tragedy, ill health, loss of a job, or accidents | .76 | .00 | .70 | .05 | .70 | .07 | .71 | .06 | .66 | .11 | .67 | .07 |  |
| I have felt a racing heart, sweaty, trouble breathing, faint, or shaky | .91 | -.10 | .89 | -.07 | .91 | -.09 | .84 | -.02 | .86 | -.06 | .91 | -.03 |  |
| I have felt tense muscles, felt on edge or restless, or had trouble relaxing or trouble sleeping | .70 | .11 | .73 | .05 | .70 | .10 | .77 | .05 | .77 | .02 | .76 | .03 |  |
| I have avoided, or did not approach or enter situations about which I worry | -.04 | .92 | -.05 | .90 | -.02 | .88 | -.02 | .90 | -.02 | .93 | -.03 | .87 |  |
| I have left situations early or participated only minimally due to worries | .03 | .87 | .02 | .85 | .00 | .88 | .00 | .91 | .01 | .88 | .03 | .95 |  |
| I have spent a lot of time making decisions, putting off making decisions, or preparing for situations, due to worries | .17 | .69 | .16 | .68 | .07 | .70 | .27 | .58 | .27 | .60 | .19 | .55 |  |
| I have sought reassurance from others due to worries | .41 | .33 | .41 | .27 | .27 | .42 | .52 | .18 | .48 | .23 | .43 | .13 |  |
| I have needed help to cope with anxiety (e.g., alcohol or medications, superstitious objects) | .58 | .21 | .59 | .15 | .52 | .21 | .58 | .14 | .54 | .18 | .51 | .24 |  |

*Note*. Generalized anxiety items = items of the GAD-D; F1 = factor 1, somatic-distress dimension; F2 = factor 2, worry-avoidance dimension.

**Tabe S5**

*Confirmatory factor analyses testing the two factor-solutions derived by the exploratory analyses in the 30% test and full sample*

| Sample | RMSEA  (≤ .05) | | RMSEA  (90% CI) | | TLI  (≥ .95) | | CFI  (≥ 0.95) | | SRMR  (≤ .08) |
| --- | --- | --- | --- | --- | --- | --- | --- | --- | --- |
| *wave 1* | |  | |  | |  | |  | |
| 30% test | .038 | | (.320, .440) | | .998 | | .998 | | .026 |
| full | .041 | | (.320, .440) | | .997 | | .998 | | .026 |
| *wave 2* |  | |  | |  | |  | |  |
| 30% test | .037 | | (.029, .046) | | .998 | | .998 | | .027 |
| full | .040 | | (.036, .044) | | .997 | | .998 | | .026 |
| *wave 3* |  | |  | |  | |  | |  |
| 30% test | .048 | | (.040, .057) | | .997 | | .997 | | .035 |
| full | .044 | | (.040, .049) | | .997 | | .998 | | .028 |
| wave 3* |  | |  | |  | |  | |  |
| 30% test | .043 | | (.034, .51) | | .997 | | .998 | | .030 |
| full | .044 | | (.039, .48) | | .997 | | .998 | | .027 |
| *wave 4* |  | |  | |  | |  | |  |
| 30% test | .032 | | (.021, .042) | | .999 | | .999 | | .025 |
| full | .037 | | (.032, .042) | | .998 | | .999 | | .023 |
| *wave 5* |  | |  | |  | |  | |  |
| 30% test | .039 | | (.030, .49) | | .997 | | .999 | | .028 |
| full | .043 | | (.38, .48) | | .997 | | .998 | | .026 |
| *wave 6* |  | |  | |  | |  | |  |
| 30% test | .043 | | (.038, .050) | | .997 | | .998 | | .030 |
| full | .045 | | (.041, .048) | | .997 | | .998 | | .028 |

*Note*. RMSEA = root mean square error of approximation; SRMR = standardized root mean square residuals; TLI = Tucker–Lewis fit index; CFI = Comparative fit index. The cut off for good fit for each fit index is printed in the header; wave 3* test of the same factor structure that was derived for the other five waves.

**C. Twin models of the somatic-distress dimension**

**Table S6**

*Fit comparisons for univariate twin models of the somatic-distress dimension*

| Base Model | Comparison Model | -2LL | df | AIC | Δ -2LL | Δ df | *p* |
| --- | --- | --- | --- | --- | --- | --- | --- |
| *Somatic-distress dimension wave 1* | | | | | | | |
| Saturated | - | 26304.55 | 8391 | 26324.55 | N/A | N/A | N/A |
| Saturated | ACE | 26315.27 | 8397 | 26323.27 | 10.72 | 6 | .10 |
| ACE | AE | 26315.27 | 8398 | 26321.27 | 0.00 | 1 | 1 |
| Saturated | **ADE** | **26310.26** | **8397** | **26318.26** | **5.71** | **6** | **.46** |
| ADE | AE | 26315.27 | 8398 | 26321.27 | 5.01 | 1 | .03 |
| *Somatic-distress dimension wave 2* | | | | | | | |
| Saturated | - | 14788.82 | 4875 | 14808.82 | N/A | N/A | N/A |
| Saturated | ACE | 14796.03 | 4881 | 14804.03 | 7.22 | 6 | .30 |
| ACE | **AE** | **14796.03** | **4882** | **14802.03** | **0.00** | **1** | **1** |
| Saturated | ADE | 14796.01 | 4881 | 14804.01 | 7.19 | 6 | .30 |
| ADE | AE | 14796.03 | 4882 | 14802.03 | 0.02 | 1 | .88 |
| *Somatic-distress dimension wave 3* | | | | | | | |
| Saturated | - | 12350.33 | 4028 | 12370.33 | N/A | N/A | N/A |
| Saturated | ACE | 12361.39 | 4034 | 12369.39 | 11.05 | 6 | .09 |
| ACE | **AE** | **12361.45** | **4035** | **12367.45** | **0.07** | **1** | **.80** |
| Saturated | ADE | 12361.45 | 4034 | 12369.45 | 11.12 | 6 | .08 |
| ADE | AE | 12361.45 | 4035 | 12367.45 | 0.00 | 1 | 1 |
| *Somatic-distress dimension wave 4* | | | | | | | |
| Saturated | - | 11224.52 | 3638 | 11244.52 | N/A | N/A | N/A |
| Saturated | ACE | 11240.77 | 3644 | 11248.77 | 16.26 | 6 | .01 |
| ACE | **AE** | **11240.77** | **3645** | **11246.77** | **0.00** | **1** | **1** |
| Saturated | ADE | 11239.26 | 3644 | 11247.26 | 14.75 | 6 | .02 |
| ADE | AE | 11240.77 | 3645 | 11246.77 | 1.51 | 1 | .22 |
| *Somatic-distress dimension wave 5* | | | | | | | |
| Saturated | - | 11852.93 | 3863 | 11872.93 | N/A | N/A | N/A |
| Saturated | ACE | 11865.19 | 3869 | 11873.19 | 12.23 | 6 | .06 |
| ACE | **AE** | **11865.19** | **3870** | **11871.19** | **0.00** | **1** | **1** |
| Saturated | ADE | 11864.54 | 3869 | 11872.54 | 11.61 | 6 | .07 |
| ADE | AE | 11865.19 | 3870 | 11871.19 | 0.65 | 1 | .42 |
| *Somatic-distress dimension wave 6* | | | | | | | |
| Saturated | - | 25051.04 | 8073 | 25071.04 | N/A | N/A | N/A |
| Saturated | ACE | 25058.48 | 8079 | 25066.48 | 7.44 | 6 | .28 |
| ACE | AE | **25058.48** | **8080** | **25064.48** | **0.00** | **1** | **1** |
| Saturated | ADE | 25056.35 | 8079 | 25064.35 | 5.31 | 6 | .50 |
| ADE | AE | 25058.48 | 8080 | 25064.48 | 2.13 | 1 | 1.4 |

*Note.* -2LL = minus twice the log likelihood; df = degrees of freedom; AIC = Akaike’s information criterion; A = additive genetic factors; C= shared environmental factors; E = non-shared environmental factors; D = dominance genetic factors. The best fitting genetic model is indicated in bold.

**Table S7**

*Twin correlations and parameter estimates with 95% confidence intervals for univariate twin models of the somatic distress dimension*

|  | rMZ | rDZ | A | D | E |
| --- | --- | --- | --- | --- | --- |
| Somatic-distress dimension wave 1 | .41  (.36 - .45) | .15  (.10 - .19) | .19  (.02 - .37) | .22  (.04 - .41) | .59  (.54 - .63) |
| Somatic-distress dimension wave 2 | .45  (.39 - .51) | .22  (.15 - .28) | .46  (.40 - .51) | - | .54  (.49 - .60) |
| Somatic-distress dimension wave 3 | .39  (.32 - .46) | .19  (.12 - .26) | .39  (.33 - .45) | - | .61  (.55 - .67) |
| Somatic-distress dimension wave 4 | .48  (.41 - .55) | .19  (.11 - .27) | .47  (.40 - .53) | - | .53  (.47 - .60) |
| Somatic-distress dimension wave 5 | .44  (.37 - .51) | .20  (.12 - .28) | .45  (.38 - .50) | - | .55  (.50 - .62) |
| Somatic-distress dimension wave 6 | .44  (.39 - .49) | .18  (.13 - .22) | .43  (.39 - .47) | - | .57  (.53 - .61) |

*Note.* rMZ = cross-twin correlations for monozygotic twins; rDZ = cross-twin correlations for dizygotic twins; A = additive genetic factors; D = dominance genetic factors; E = non-shared environmental factors; somatic-distress dimension wave 1 to 6 = square root transformed total score of the items loading onto the somatic-distress factor of the GAD-D, wave 1 to 6. Parameter estimates presented in the table are variance components. To obtain path coefficients, estimates should be square rooted.

**Table S8**

*Fit comparisons for multivariate twin models of the somatic-distress dimension*

| Base Model | Comparison Model | -2LL | df | AIC | Δ -2LL | Δ df | *p* |
| --- | --- | --- | --- | --- | --- | --- | --- |
| Saturated | - | 86315.93 | 32748 | 86675.93 | N/A | N/A | N/A |
| Saturated | Constrained | 86474.65 | 32859 | 86612.65 | 158.72 | 111 | <.01 |
| *Cholesky Decomposition* | | |  |  |  |  |  |
| Saturated | ACE | 86490.49 | 32859 | 86628.49 | 174.56 | 111 | <.001 |
| Saturated | AE | 86492.90 | 32880 | 86588.90 | 176.97 | 132 | <.01 |
| ACE | AE | 86492.90 | 32880 | 86588.90 | 2.41 | 21 | 1 |
| Saturated | ADE | 86484.14 | 32859 | 86622.14 | 168.21 | 111 | <.001 |
| ADE | **AE** | **86492.90** | **32880** | **86588.90** | **8.75** | **21** | **.99** |
| *Common pathway* | |  |  |  |  |  |  |
| Saturated | ACE | 86650.80 | 32896 | 86716.80 | 334.88 | 148 | <.001 |
| Saturated | AE | 86651.27 | 32903 | 86703.27 | 335.34 | 155 | <.001 |
| ACE | AE | 86651.27 | 32903 | 86703.27 | 0.46 | 7 | 1 |
| Saturated | ADE | 86645.96 | 32896 | 86711.96 | 330.03 | 148 | <.001 |
| ADE | **AE** | **86651.27** | **32903** | **86703.27** | **5.30** | **7** | **.62** |

*Note*. -2LL = minus twice the log likelihood; df = degrees of freedom; AIC = Akaike’s information criterion; A = additive genetic factors; C= shared environmental factors; E = non-shared environmental factors; D = dominance genetic factors; constrained = constrained model with equal means and variances across twins and zygosity groups as well as symmetric cross-twin cross-wave covariance matrices. The best fitting Cholesky and common pathway model is indicated in bold. The best fitting Cholesky and common pathway models are indicated in bold. Fit of the best fitting Cholesky and common pathway model was significantly worse than fit of the saturated model. However, this is common in twin studies with large sample sizes, where minimal variance deviations from the model’s assumptions can be statistically significant (e.g., Waszczuk et al., 2016; Cheesman et al., 2018).

**Table S9**

|  | w1 factors | | w2 factors | | w3 factors | | w4 factors | | w5 factors | | w6 factors | |
| --- | --- | --- | --- | --- | --- | --- | --- | --- | --- | --- | --- | --- |
|  | A_1_ | E_1_ | A_2_ | E_2_ | A_3_ | E_3_ | A_4_ | E_4_ | A_5_ | E_5_ | A_6_ | E_6_ |
| Somatic-distress dimension wave 1 | **.40**  (.36-.43) | **.60**  (.57-.64) |  |  |  |  |  |  |  |  |  |  |
| Somatic-distress dimension wave 2 | **.34**  (.29-.40) | **.08**  (.06 -.11) | **.11**  (.06-.16) | **.47**  (.43-.51) |  |  |  |  |  |  |  |  |
| Somatic-distress dimension wave 3 | **.37**  (.32-.43) | **.08**  (.06-.11) | .03  (.00-.07) | **.14**  (.11-.18) | .02  (.00-.04) | **.36**  (.33-.39) |  |  |  |  |  |  |
| Somatic-distress dimension wave 4 | **.38**  (.32-.44) | **.08**  (.05-.10) | **.04**  (.01-.08) | **.09**  (.07-.12) | .02  (.00-.06) | **.05**  (.03-.07) | .02  (.00-.05) | **.33**  (.30-.36) |  |  |  |  |
| Somatic-distress dimension wave 5 | **.37**  (.32-.43) | **.07**  (.05-.10) | **.05**  (.01-.10) | **.07**  (.05-.10) | .00  (.00-.04) | **.04**  (.03-.06) | .01  (.00-.04) | **.03**  (.02-.04) | .00  (.00-.03) | **.34**  (.31-.36) |  |  |
| Somatic-distress dimension wave 6 | **.38**  (.33-.43) | **.06**  (.04-.08) | .02  (.00-.06) | **.05**  (.03-.06] | .00  (.00-.06) | **.03**  (.02-.04) | .04  (.00-.08) | **.02**  (.01-.03) | .00  (.00-.06) | **.02**  (.01-.03) | .00  (.00-.05) | **.38**  (.35-.41) |

*Parameter estimates with 95% confidence intervals for Cholesky decomposition of the somatic-distress dimension*

*Note*. A = additive genetic factors; E = non-shared environmental factors; somatic-distress dimension wave 1 to 6 = square root transformed total score of the items loading onto the somatic-distress factor of the GAD-D, wave 1 to 6. Parameter estimates presented in the table are variance components. To obtain path coefficients, estimates should be square rooted. Significant estimates are indicated in bold.

**Figure S1**

*Common pathway model of the somatic-distress dimension*


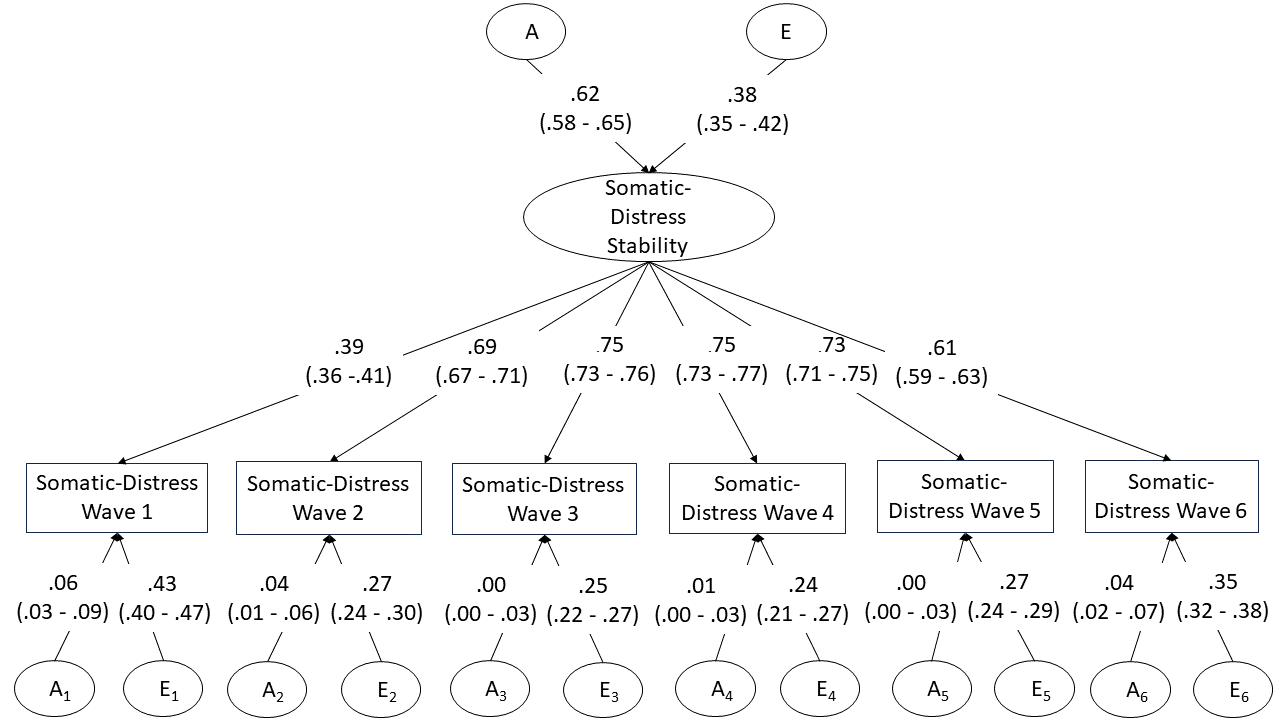


*Note*. A = additive genetic factors; E = non-shared environmental factors; somatic-distress wave 1 to 6 = square root transformed total score of the items loading onto the somatic-distress factor of GAD-D, wave 1 to 6; somatic-distress stability = latent stability of the somatic-distress dimension. Parameter estimates presented in the figure are variance components. To obtain path coefficients, estimates should be square rooted.

**C. Twin models of the worry-avoidance dimension**

**Table S10**

*Fit comparisons for univariate twin models of the worry-avoidance dimension*

| Base Model | Comparison Model | -2LL | df | AIC | Δ -2LL | Δ df | *p* |
| --- | --- | --- | --- | --- | --- | --- | --- |
| *Worry-avoidance dimension wave 1* | | | | | | | |
| Saturated | - | 23219.81 | 8391 | 23239.81 | N/A | N/A | N/A |
| Saturated | ACE | 23227.86 | 8397 | 23235.86 | 8.05 | 6 | .23 |
| ACE | **AE** | **23227.86** | **8398** | **23233.86** | **0.00** | **1** | **1** |
| Saturated | ADE | 23225.55 | 8397 | 23233.55 | 5.74 | 6 | .45 |
| ADE | AE | 23227.86 | 8398 | 23233.86 | 2.32 | 1 | 1.3 |
| *Worry-avoidance dimension wave 2* | | | | | | | |
| Saturated | - | 13497.27 | 4875 | 13517.27 | N/A | N/A | N/A |
| Saturated | ACE | 13502.09 | 4881 | 13510.09 | 4.81 | 6 | .57 |
| ACE | **AE** | **13502.09** | **4882** | **13508.09** | **0.00** | **1** | **1** |
| Saturated | ADE | 13502.00 | 4881 | 13510.00 | 4.73 | 6 | .58 |
| ADE | AE | 13502.09 | 4882 | 13508.09 | 0.08 | 1 | .77 |
| *Worry-avoidance dimension wave 3* | | | | | | | |
| Saturated | - | 11215.01 | 4028 | 11235.01 | N/A | N/A | N/A |
| Saturated | ACE | 11221.95 | 4034 | 11229.95 | 6.94 | 6 | .33 |
| ACE | **AE** | **11223.13** | **4035** | **11229.13** | **1.18** | **1** | **2.78** |
| Saturated | ADE | 11223.13 | 4034 | 11231.13 | 8.12 | 6 | .23 |
| ADE | AE | 11223.13 | 4035 | 11229.13 | 0.00 | 1 | 1 |
| *Worry-avoidance dimension wave 4* | | | | | | | |
| Saturated | - | 10171.40 | 3638 | 10191.40 | N/A | N/A | N/A |
| Saturated | ACE | 10176.47 | 3644 | 10184.47 | 5.07 | 6 | .53 |
| ACE | **AE** | **10176.47** | **3645** | **10182.47** | **0.00** | **1** | **1** |
| Saturated | ADE | 10176.44 | 3644 | 10184.44 | 5.03 | 6 | .54 |
| ADE | AE | 10176.47 | 3645 | 10182.47 | 0.03 | 1 | .85 |
| *Worry-avoidance dimension wave 5* | | | | | | | |
| Saturated | - | 10703.92 | 3863 | 10723.92 | N/A | N/A | N/A |
| Saturated | ACE | 10706.75 | 3869 | 10714.75 | 2.84 | 6 | .83 |
| ACE | **AE** | **10709.65** | **3870** | **10715.65** | **2.90** | **1** | **.09** |
| Saturated | ADE | 10709.65 | 3869 | 10717.65 | 5.73 | 6 | .45 |
| ADE | AE | 10709.65 | 3870 | 10715.65 | 0.00 | 1 | 1 |
| *Worry-avoidance dimension wave 6* | | | | | | | |
| Saturated | - | 22734.15 | 8095 | 22754.15 | N/A | N/A | N/A |
| Saturated | ACE | 22738.14 | 8101 | 22746.14 | 3.99 | 6 | .68 |
| ACE | **AE** | **22738.14** | **8102** | **22744.14** | **0.00** | **1** | **1** |
| Saturated | ADE | 22738.09 | 8101 | 22746.09 | 3.94 | 6 | .68 |
| ADE | AE | 22738.14 | 8102 | 22744.14 | 0.05 | 1 | .83 |

*Note.* -2LL = minus twice the log likelihood; df = degrees of freedom; AIC = Akaike’s information criterion; A = additive genetic factors; C= shared environmental factors; E = non-shared environmental factors; D = dominance genetic factors. The best fitting genetic model is indicated in bold.

**Table S11**

*Twin correlations and parameter estimates with 95% confidence intervals for univariate twin models of the worry-avoidance dimension*

|  | rMZ | rDZ | A | E |
| --- | --- | --- | --- | --- |
| Worry-avoidance dimension wave 1 | .31  (.26 - .35) | .11  (.07 - .16) | .29  (.25 - .33) | .71  (.67 - .75) |
| Worry-avoidance dimension wave 2 | .33  (.26 - .40) | .15  (.09 - .22) | .33  (.27 - .39) | .67  (.61 - .83) |
| Worry-avoidance dimension wave 3 | .28  (.20 - .35) | .17  (.10 - .24) | .29  (.22 - .35) | .71  (.65 - .78) |
| Worry-avoidance dimension wave 4 | .32  (.23 - .39) | .15  (.07 - .22) | .31  (.24 - .38) | .69  (.62 - .76) |
| Worry-avoidance dimension wave 5 | .34  (.26 - .41) | .25  (.17 - .32) | .37  (.31 - .43) | .63  (.57 - .69) |
| Worry-avoidance dimension wave 6 | .35  (.30- .40) | .16  (.12 - .21) | .35  (.31 - .49) | .65  (.60 - .69) |

*Note.* rMZ = cross-twin correlations for monozygotic twins; rDZ = cross-twin correlations for dizygotic twins; A = additive genetic factors; D = dominance genetic factors; E = non-shared environmental factors; worry-avoidance dimension wave 1 to 6 = square root transformed total score of the items loading onto the worry-avoidance factor of the GAD-D, wave 1 to 6. Parameter estimates presented in the table are variance components. To obtain path coefficients, estimates should be square rooted.

**Table S12**

*Fit comparisons for multivariate twin models of the worry-avoidance dimension*

| Base Model | Comparison Model | -2LL | df | AIC | Δ -2LL | Δ df | *p* |
| --- | --- | --- | --- | --- | --- | --- | --- |
| Saturated | - | 81045.85 | 32770 | 81405.85 | N/A | N/A | N/A |
| Saturated | Constrained | 81161.70 | 32881 | 81299.70 | 115.85 | 111 | .36 |
| *Cholesky Decomposition* | | | | | | |  |
| Saturated | ACE | 81176.68 | 32881 | 81314.68 | 130.82 | 111 | .10 |
| Saturated | AE | 81180.33 | 32902 | 81276.33 | 134.47 | 132 | .42 |
| ACE | AE | 81180.33 | 32902 | 81276.33 | 3.65 | 21 | 1 |
| Saturated | ADE | 81177.09 | 32881 | 81315.09 | 31.23 | 111 | .09 |
| ADE | **AE** | **81180.33** | **32902** | **81276.33** | **3.24** | **21** | **1** |
| *Common pathway* | |  |  |  |  |  |  |
| Saturated | ACE | 81298.23 | 32918 | 81364.23 | 252.37 | 148 | <.001 |
| Saturated | AE | 81299.74 | 32925 | 81351.74 | 253.89 | 155 | <.001 |
| ACE | AE | 81299.74 | 32925 | 81351.74 | 1.52 | 7 | .98 |
| Saturated | ADE | 81296.63 | 32918 | 81362.63 | 250.78 | 148 | <.001 |
| ADE | **AE** | **81299.74** | **32925** | **81351.74** | **3.12** | **7** | **.87** |

*Note*. -2LL = minus twice the log likelihood; df = degrees of freedom; AIC = Akaike’s information criterion; A = additive genetic factors; C= shared environmental factors; E = non-shared environmental factors; D = dominance genetic factors; constrained = constrained model with equal means and variances across twins and zygosity groups as well as symmetric cross-twin cross-wave covariance matrices. The best fitting Cholesky and common pathway model is indicated in bold. The best fitting common pathway models are indicated in bold. Fit of the best fitting Cholesky and common pathway model was significantly worse than fit of the saturated model. However, this is common in twin studies with large sample sizes, where minimal variance deviations from the model’s assumptions can be statistically significant (e.g., Waszczuk et al., 2016; Cheesman et al., 2018).

**Table S13**

|  | w1 factors | | w2 factors | | w3 factors | | w4 factors | | w5 factors | | w6 factors | | |
| --- | --- | --- | --- | --- | --- | --- | --- | --- | --- | --- | --- | --- | --- |
|  | A_1_ | E_1_ | A_2_ | E_2_ | A_3_ | E_3_ | A_4_ | E_4_ | A_5_ | E_5_ | A_6_ | E_6_ |  |
| Worry-avoidance dimension wave 1 | **.29**  (.25-.23) | **.71**  (.67-.75) |  |  |  |  |  |  |  |  |  |  |  |
| Worry-avoidance dimension wave 2 | **.24**  (.18-.30) | **.06**  (.04-.08) | **.09**  (.03-.14) | **.61**  (.57-.67) |  |  |  |  |  |  |  |  |  |
| Worry-avoidance dimension wave 3 | **.24**  (.18-.30) | **.06**  (.04-.09) | **.07**  (.02-.12) | **.09**  (.07-.12) | .00  (.00-.03) | **.54**  (.50-.57) |  |  |  |  |  |  |  |
| Worry-avoidance dimension wave 4 | **.28**  (.22-.34) | **.05**  (.03-.07) | .02  (.00-.06) | **.08**  (.06-.11) | .00  (.00-.04) | **.07**  (.05-.09) | .01  (.00-.04) | **.49**  (.45-.52) |  |  |  |  |  |
| Worry-avoidance dimension wave 5 | **.25**  (.19-.31) | **.06**  (.04-.08) | **.07**  (.02-.13) | **.06**  (.04-.08) | .00  (.00-.08) | **.05**  (.04-.07) | .03  (.00-.08) | **.05**  (.03-.07) | .00  (.00-.06) | **.43**  (.39-.67) |  |  |  |
| Worry-avoidance dimension wave 6 | **.25**  (.20-.31) | **.06**  (.04-.08) | .03  (.00-.09) | **.04**  (.02-.05) | .01  (.00-.01) | **.03**  (.02-.05) | .06  (.00-.11) | **.02**  (.01-.04) | .00  (.00-.08] | **.02**  (.01-.03) | .00  (.00-.06) | **.48**  (.44-.51) |  |

*Parameter estimates with 95% confidence intervals for Cholesky decomposition of the worry-avoidance dimension*

*Note*. A = additive genetic factors; E = non-shared environmental factors; worry-avoidance dimension wave 1 to 6 = square root transformed total score of the items loading onto the worry-avoidance factor of the GAD-D, wave 1 to 6. Parameter estimates presented in the table are variance components. To obtain path coefficients, estimates should be square rooted. Significant estimates are indicated in bold.

**Figure S2**

*Common pathway model of the worry-avoidance dimension*


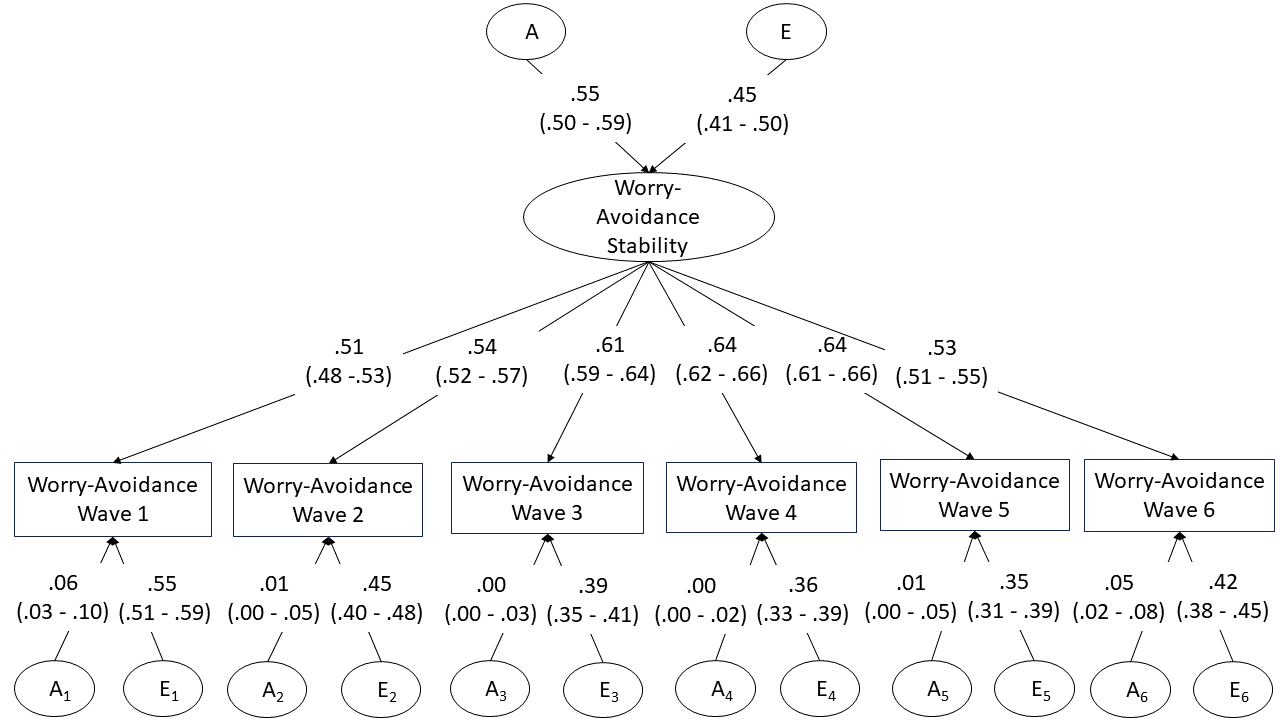


*Note*. A = additive genetic factors; E = non-shared environmental factors; worry-avoidance wave 1 to 6 = square root transformed total score of the items loading onto the worry-avoidance factor of the GAD-D, wave 1 to 6; worry-avoidance stability = latent stability of the worry-avoidance dimension. Parameter estimates presented in the figure are variance components. To obtain path coefficients, estimates should be square rooted.

**Table S14**

*Fit comparisons for multivariate twin models of somatic-distress and worry-avoidance dimension*

| Base Model | Comparison Model | -2LL | df | AIC | Δ -2LL | Δ df | *p* |
| --- | --- | --- | --- | --- | --- | --- | --- |
| Saturated | - | 82347.71 | 32902 | 82523.71 | N/A | N/A | N/A |
| Saturated | Constrained | 82380.90 | 32932 | 82496.90 | 3.19 | 30 | .31 |
| *Cholesky decomposition (correlated factor model)* | | | | | | | |
| Saturated | ACE | 82409.84 | 32956 | 82477.84 | 62.13 | 54 | .21 |
| Saturated | **AE** | **82410.90** | **32966** | **82458.90** | **63.18** | **64** | **.51** |
| ACE | AE | 82410.90 | 32966 | 82458.90 | 1.054 | 10 | 1 |
| Saturated | ADE | 82401.65 | 32956 | 82469.65 | 53.93 | 54 | .48 |
| ADE | AE | 82410.90 | 32966 | 82458.90 | 9.25 | 10 | .51 |

*Note*. -2LL = minus twice the log likelihood; df = degrees of freedom; AIC = Akaike’s information criterion; A = additive genetic factors; C= shared environmental factors; E = non-shared environmental factors; D = dominance genetic factors. The best fitting Cholesky (correlated factor) model is indicated in bold.

**Figure S3**

*Correlated factor solution of somatic-distress and worry-avoidance dimension*

*
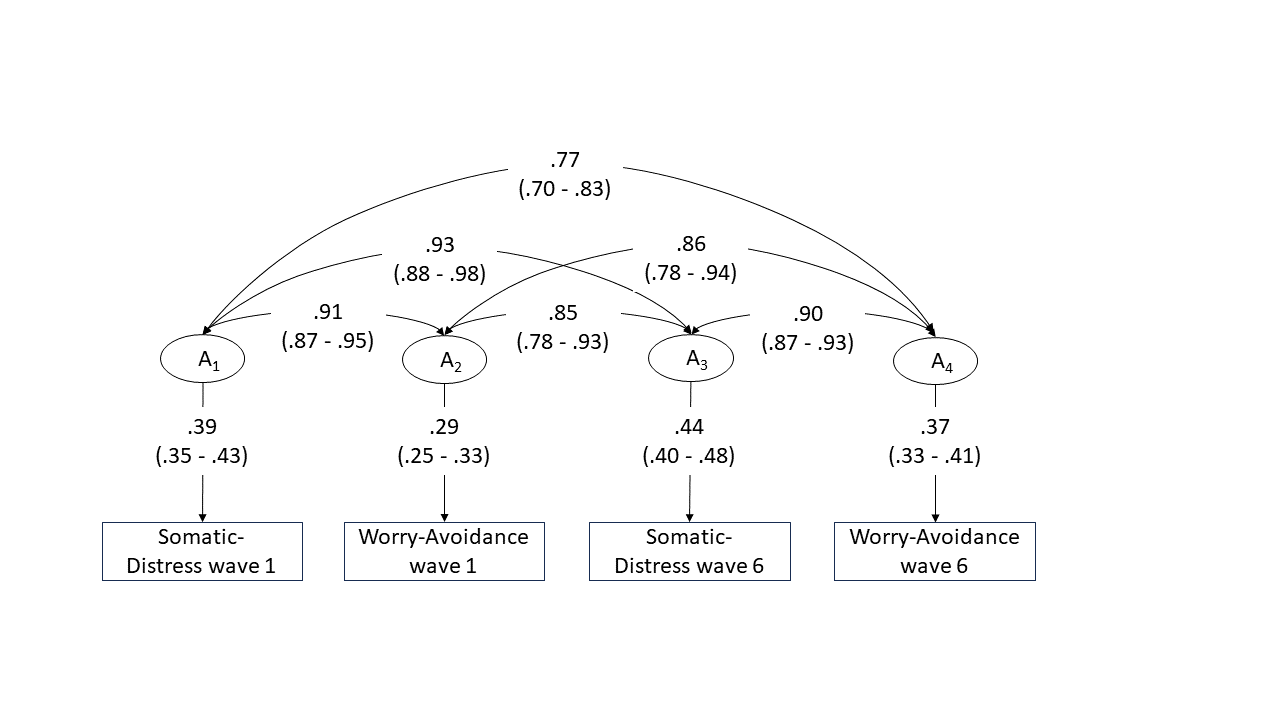
*


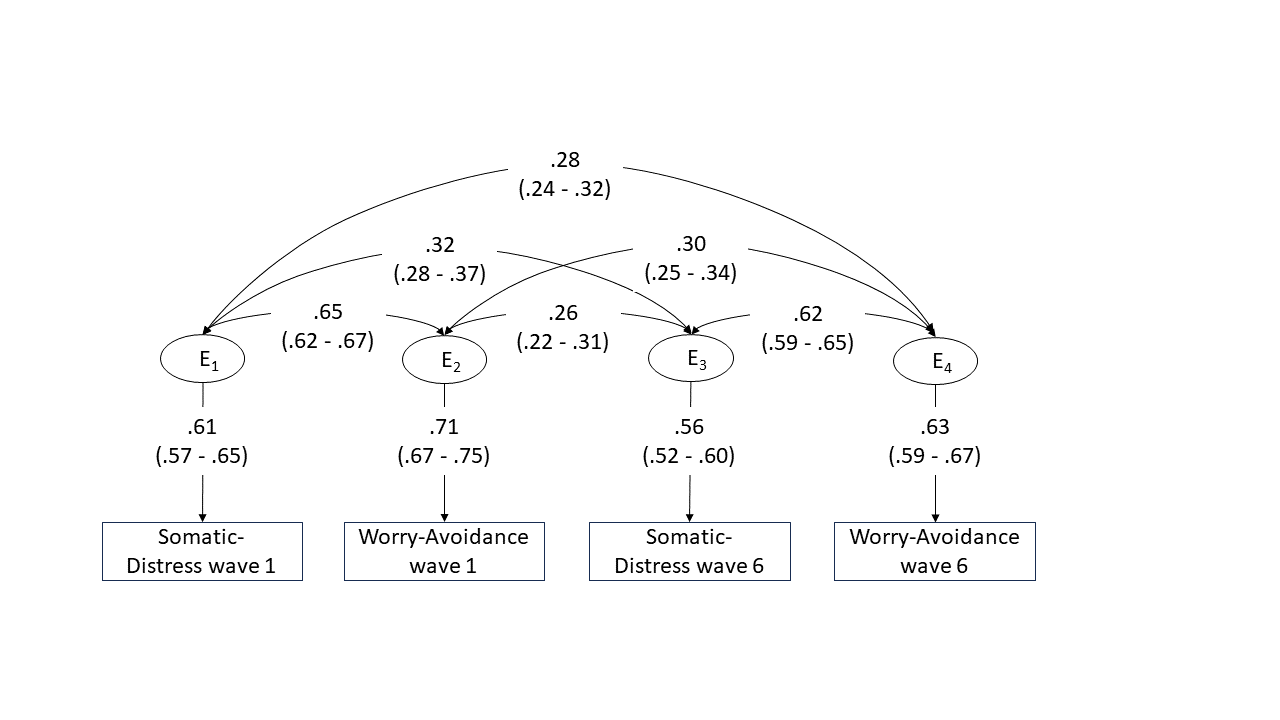


*Note*. A = additive genetic factors; E = non-shared environmental factors; worry-avoidance wave 1 and 6 = square root transformed total score of the items loading onto the worry-avoidance factor of the GAD-D, wave 1 and 6; somatic-distress wave 1 and 6 = square root transformed total score of the items loading onto the somatic-distress factor of the GAD-D, wave 1 and 6. For reasons of clarity, the correlated factor solution was estimated based on data from wave 1 and 6 only.
